# Digital Health Literacy and Health Technology Readiness Among People With Epilepsy or Multiple Sclerosis: Cross-Sectional Study

**DOI:** 10.2196/85625

**Published:** 2026-03-05

**Authors:** Anna Vahteristo, Virpi Jylhä, Hanna Kuusisto

**Affiliations:** 1 Department of Health and Social Management, University of Eastern Finland, P.O. Box 1627, Kuopio, 70211, Finland, 358 0504156223; 2 Research Centre for Nursing Science and Social and Health Management, Wellbeing Services County of North Savo, Kuopio University Hospital, Kuopio, Finland; 3 Department of Neurology, Tampere University Hospital, Tampere, Finland

**Keywords:** digital health, digital health literacy, health technology readiness, epilepsy, multiple sclerosis

## Abstract

**Background:**

Digital health services (DHS) are an increasingly integral part of health care services. Understanding users’ abilities to engage with DHS is crucial to ensuring that health technology meets their needs. Assessing digital health literacy (DHL) and health technology readiness can help identify the strengths and weaknesses of DHL in different subgroups.

**Objective:**

This study aimed to assess DHL and health technology readiness among people with epilepsy or multiple sclerosis (MS) and, accordingly, identify and categorize them into distinct subgroups or profiles. In addition, we aimed to investigate respondents’ use of DHS in managing their chronic condition and differences in DHL and health technology readiness between DHS users and nonusers.

**Methods:**

An electronic survey was distributed to people with epilepsy or MS with the help of patient organizations. The questionnaire included the Finnish version of the Readiness and Enablement Index for Health Technology. The subgroups of respondents were identified using k-means cluster analysis. Nonparametric tests were used to compare health technology readiness among identified subgroups.

**Results:**

Respondents (N=289) had mid- to high-level scores in all the dimensions describing DHL and health technology readiness. A total of 4 distinct profiles emerged with different strengths and weaknesses in their DHL and health technology readiness. There was a significantly higher proportion of DHS users among the 2 profiles with the highest DHL, profile 1 (62/81, 76.5%) and profile 2 (59/80, 74.7%), compared with the profile with the lowest DHL, profile 4 (20/50, 40%; *P*<.001). In contrast, those with the lowest confidence in their DHL had higher emotional distress, reported lower confidence in the support from their health care providers, and had a smaller proportion of DHS users. In addition, the DHS users had significantly higher DHL levels in 6 of the 7 dimensions, as well as higher confidence in the support they received from their health care providers (mean 2.71, SD 0.72; *P*=.01) compared with nonusers (mean 2.42, SD 0.90) and in social support for health (mean 2.81, SD 0.71; *P*=.02), compared with nonusers (mean 2.54, SD 0.85).

**Conclusions:**

Identifying subgroups with distinct profiles, characterized by different strengths and weaknesses in their DHL and health technology readiness, is crucial in ensuring the development of responsive and inclusive DHS to meet the needs of all users, particularly those requiring support in using DHS. In addition, the nonusers had lower confidence in the support they received from their health care provider than the users. Further research is needed to understand this difference.

## Introduction

Digital health interventions have proven to be clinically effective [1-9], cost-effective [2,3], and beneficial in the self-management of chronic diseases [4,10]. These interventions are closely associated with digital health literacy (DHL) [11-14], which refers to the ability to search for, understand, and evaluate health-related information from electronic sources and then use it to solve health-related problems [15]. Therefore, assessing DHL is crucial for elucidating the success of digital health interventions [16].

A high DHL level has been associated with higher education [17,18], increased use of digital health [17,19,20], and digital competence [21]. However, the relationship between DHL and chronic diseases remains complex. Some studies have noted low or inadequate DHL among patients with chronic diseases [17,22,23], whereas others have found good levels of DHL [18,19]. Chronic diseases, including neurological disorders [24], are a growing cause of increased disability-adjusted life years and a burden on health care systems [25]. Epilepsy and multiple sclerosis (MS) are chronic preference-sensitive neurologic conditions [26] with multiple treatment options [27,28]. Treatment success depends on a combination of evidence-based medicine and patient-provided information [29] alongside individualized care plans and regular monitoring [30,31].

Both patients and health care providers have found digital health applications useful in the care and self-management of chronic conditions [32]. This trend is reflected in the increasing use of digital health technology for neurological care [33]. For example, the Finnish Neuro Registry is integrated into electronic patient records and includes a patient interface for people with MS and a digital seizure diary for those with epilepsy [34]. Similarly, electronic patient interfaces for epilepsy [35] and MS [36], as well as disease-specific digital care pathways for MS [37,38], have demonstrated promising results in improving care [35-37].

To ensure the effectiveness of digital health services (DHS), it is essential to elucidate the DHL of individuals with chronic diseases. In addition to disease-related background and sociodemographic characteristics, individuals’ health technology readiness and emotional and mental well-being should be assessed to identify vulnerable subgroups [39]. It is also essential to understand more about individuals’ strengths and weaknesses in terms of DHL and health technology use. Therefore, in this study, we assessed DHL and health technology readiness among people with epilepsy or MS and identified profiles of DHL and health technology readiness in this population. In addition, we investigated respondents’ use of DHS in the management of their chronic condition and compared DHL and health technology readiness between the users and nonusers of DHS.

## Methods

### Recruitment

A cross-sectional survey was conducted between April 2022 and April 2023 to investigate DHL and health technology readiness among people with epilepsy or MS. The questionnaire was available in both Finnish and Swedish, the official languages of Finland. The survey targeted people diagnosed with epilepsy or MS, with no other eligibility criteria.

The first round of data collection began in April 2022 with an invitation to an online survey distributed by the Finnish Neuro Society to its members. The survey was closed in August 2022, when no further responses were received despite reminders. The second round involved paper questionnaires and the online survey. The latter was distributed by the Finnish Epilepsy Association to its members in November 2022 and by the Finnish Pensioners’ Federation in January 2023, with the aim of reaching members diagnosed with epilepsy. The second round of data collection was closed in March 2023. The paper questionnaires were distributed to all eligible patients at the Department of Neurology of a university hospital in Finland. Additionally, they were distributed at events organized by the Finnish Epilepsy Association. The final paper questionnaires were distributed at the end of March, with a final response deadline in April 2023.

### Instruments

**Figure 1.**
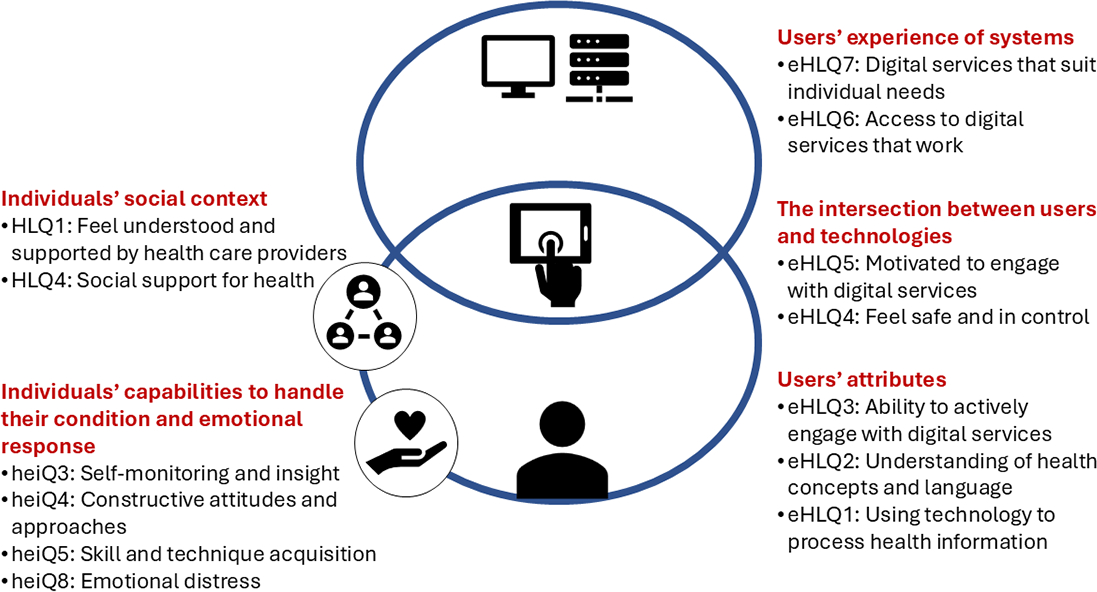
Finnish version of the Readiness and Enablement Index for Health Technology [40] (adapted from Kayser et al [41], which is published under Creative Commons Attribution 4.0 International License [46]). eHLQ: eHealth Literacy Questionnaire; heiQ: Health Education Impact Questionnaire; HLQ: Health Literacy Questionnaire.

The questionnaire was based on the Finnish version of the Readiness and Enablement Index for Health Technology (READHY-FIN) [40]. Readiness and Enablement Index for Health Technology (READHY) is an internationally validated instrument that measures levels of DHL and health technology readiness [41]. It consists of questions from 3 widely used instruments: the Health Education Impact Questionnaire [42], the Health Literacy Questionnaire [43], and the eHealth Literacy Questionnaire [44]. READHY-FIN comprises 65 items across 13 dimensions assessing 5 aspects of DHL and health technology readiness (Figure 1 [41]). These items are rated on a 4-point Likert scale ranging from 1=“strongly disagree” to 4=“strongly agree.” The dimension of *emotional distress* was reverse-coded such that a higher score depicted less emotional distress. The score for each dimension and domain was calculated as the mean of the scores for the included items. If at least 50% of the items within a dimension were answered, the score for the dimension was manually calculated as an average of the items that were answered [41]. The READHY-FIN instrument was translated and culturally tested according to the protocol of the University of Swinburne [45] and validated in the Finnish population [40]. For the Swedish questionnaire, we used the Swedish version of READHY (READHY-SWE), which was translated by the developers of the original instrument [41].

Additionally, the questionnaire included items describing the participants’ sociodemographic and disease characteristics. The use of different service channels for managing their chronic condition included multiple choices. Of these, the use of DHS was defined as the use of any digital health service channel (patient interfaces or digital care pathways for MS or epilepsy) to manage their chronic condition; nonusers were defined as those who never used DHS to manage their chronic condition.

### Statistical Analysis

The data analysis was performed with SPSS (version 29.0; IBM Corp). The data are presented as percentages and means. K-means clustering was used to identify profiles of respondents based on the dimensions of the READHY instrument. Cluster analysis can be used to identify subgroups or profiles of technology users based on the similarity of response patterns [47]. In previous studies using the READHY instrument, the number of clusters was 4 [41,48], 5 [39], or 6 [49]. Therefore, we tested our data using models ranging from 3 to 6 clusters. We assessed the appropriateness of the number of clusters by evaluating SDs and ANOVA for between-group differences, as well as pairwise comparisons between profiles on each READHY dimension. We chose the 4-cluster model because it produced the best between-profile variability and minimal within-profile variability.

To increase understanding of the dimensions and domains of READHY in different groups of respondents, we conducted comparisons based on respondents’ characteristics, use of DHS, and the profiles identified in cluster analysis. We conducted further analyses using nonparametric Kruskal-Wallis and Mann-Whitney tests to compare different profiles and respondent characteristics, as well as to compare the characteristics of users and nonusers of DHS. The significance level was set at *P*<.05 [50]. Pairwise comparisons were performed when significant differences (*P*<.05) were indicated. Significance values were adjusted by the Bonferroni method for multiple tests to control for type I error.

### Ethical Considerations

Ethics approval for the research was obtained from the Ethics Committee of Tampere University Hospital (R21057). The research complied with the World Medical Association’s Declaration of Helsinki and was conducted in accordance with the principles of good research practice [51,52], the Finnish National Medical Research Act [53], and guidelines governing nonmedical research [54].

The respondents were informed that participation was voluntary and they had the opportunity to opt out at any time. Their anonymity was guaranteed, and no identifiable information was collected. Written informed consent was obtained from the respondents after they read the information sheet and privacy notice and before they started to fill out the anonymous questionnaire. Responding on behalf of a person was possible when the respondent was unable to do so themselves [54]. No compensation was provided to participants.

## Results

### Respondent Characteristics

The total number of participants was 289; their characteristics are summarized in Table 1. Their average age was 49.3 (SD 13.3) years, with most respondents (228/287, 79.4%) identifying as women. Two-thirds (188/289, 65.1%) of participants were diagnosed with MS, and two-thirds (188/288, 65.3%) were identified as users of DHS in managing their chronic condition.

**Table 1.** Characteristics of all respondents (N=289) and of the 4 Readiness and Enablement Index for Health Technology profiles.

Sociodemographic variables	All (N=289), n (%)	Profile 1 (n=81), n (%)	Profile 2 (n=80), n (%)	Profile 3 (n=78), n (%)	Profile 4 (n=50), n (%)
Gender (n=287)					
Woman	228 (79.4)	61 (76.3)	64 (80.0)	64 (82.1)	39 (79.6)
Man	59 (20.6)	19 (23.8)	16 (20.0)	14 (17.9)	10 (20.4)
Age groups (years; n=284)					
<30	20 (7.0)	6 (7.4)	6 (7.7)	5 (6.5)	3 (6.3)
30-39	47 (16.5)	18 (22.2)	10 (12.8)	12 (15.6)	7 (14.6)
40-54	114 (40.1)	30 (37.0)	33 (42.3)	26 (33.8)	25 (52.1)
55-64	66 (23.2)	16 (19.8)	18 (23.1)	21 (27.3)	11 (22.9)
>64	37 (13.0)	11 (13.6)	11 (14.1)	13 (16.9)	2 (4.2)
Diagnosed with chronic disease					
Epilepsy	101 (34.9)	25 (30.9)	24 (30.0)	32 (41.0)	20 (40.0)
Multiple sclerosis	188 (65.1)	56 (69.1)	56 (70.0)	46 (59.0)	30 (60.0)
Time from the diagnosis of chronic disease (years; n=287)					
<1	8 (2.8)	4 (4.9)	4 (5.0)	0 (0.0)	0 (0.0)
1-5	90 (31.4)	22 (27.2)	30 (37.5)	19 (25.0)	19 (38.0)
6-10	39 (13.6)	13 (16.0)	7 (8.8)	16 (21.1)	3 (6.0)
>10	150 (52.3)	42 (51.9)	39 (48.8)	41 (53.9)	28 (56.0)
Educational level (n=286)					
Primary and lower secondary education	22 (7.7)	6 (7.9)	5 (6.3)	5 (6.4)	6 (12.2)
Upper secondary or vocational education	88 (30.8)	16 (20.0)	21 (26.6)	26 (33.3)	25 (51.0)
Bachelor’s-level degree	89 (31.1)	24 (30.0)	28 (35.4)	26 (33.3)	11 (22.4)
Master’s-level or doctoral degree	87 (30.4)	34 (42.5)	25 (31.6)	21 (26.9)	7 (14.3)
Use of DHS^a^ for the care of chronic disease (n=288)					
Active users (monthly or more often)	58 (20.1)	20 (24.7)	18 (22.5)	15 (19.5)	5 (10.0)
Occasional users (once or few times a year)	130 (45.1)	42 (51.9)	41 (51.3)	32 (41.6)	15 (30.0)
Nonusers	100 (34.7)	19 (23.5)	21 (26.3)	30 (40.0)	30 (60.0)

a DHS: digital health services.

Most respondents were evenly distributed across 3 of the 4 educational levels (Table 1): upper secondary school or vocational education (88/288, 30.8%), lower-level university degree (89/288, 31.1%), and upper-level university degree or doctoral degree (87/288, 30.4%).

### The Use of Health Care Services and Digital Health for Managing Chronic Conditions

The use of various health care channels for managing respondents’ chronic conditions is reported in Table 2. Face-to-face visits were the most common channel used by the respondents (271/288, 94.1%). However, nearly two-thirds (172/271, 63.5%) of those with face-to-face visits contacted their health care provider only once a year or less often.

Nearly half (91/188, 48.4%) of the users of DHS for managing their chronic condition had used DHS a few times a year, and one-quarter (47/188, 25.0%) of the users had used it monthly. There were also very active users, as 5.9% (11/188) used DHS weekly.

**Table 2.** Use of health care service channels.

	Face-to-face clinical visits (n=288), n (%)	Phone and video calls (n=289), n (%)	Digital health services (n=288), n (%)
Weekly or several times a week	3 (1.0)	1 (0.3)	11 (3.8)
Monthly	7 (2.4)	6 (2.1)	47 (16.3)
Few times a year	89 (30.9)	91 (31.5)	91 (31.6)
Once a year or less often	172 (59.7)	86 (29.8)	39 (13.5)
Not at all	17 (5.9)	105 (36.3)	100 (34.7)

### DHL and Health Technology Readiness

The average level of respondents’ DHL and health technology readiness, measured using the READHY dimensions, ranged from 2.61 to 3.19 (Multimedia Appendix 1). Respondents were highly confident in their *ability to actively engage with digital services* (mean 3.19, SD 0.70), *self-monitoring and insight* (mean 3.19, SD 0.42), and *constructive attitudes and approaches* (mean 3.00, SD 0.71). The dimensions in which respondents were least confident were *feeling understood and supported by health care providers* (mean 2.61, SD 0.82) and *social support for health* (mean 2.72, SD 0.76).

Respondents with higher educational levels had more confidence in their DHL and health technology readiness than those with lower educational levels. In pairwise comparisons, participants with master-level or doctoral degrees scored significantly higher than respondents with upper secondary school or vocational education in the domains of *users’ attributes* (*P*<.001), *the intersection between users and technologies* (*P*=.001)*, users’ experience of systems* (*P*<.001), and *individuals’ social context* (*P*=.005). Additionally, respondents with bachelor-level degrees had significantly higher scores than those with upper secondary or vocational education in the domains of *intersection between users and technologies* (*P*=.02) and *users’ experience of systems* (*P*=.02).

### Four READHY Profiles to Describe Subgroups of Individuals With Epilepsy or MS

**Figure 2.**
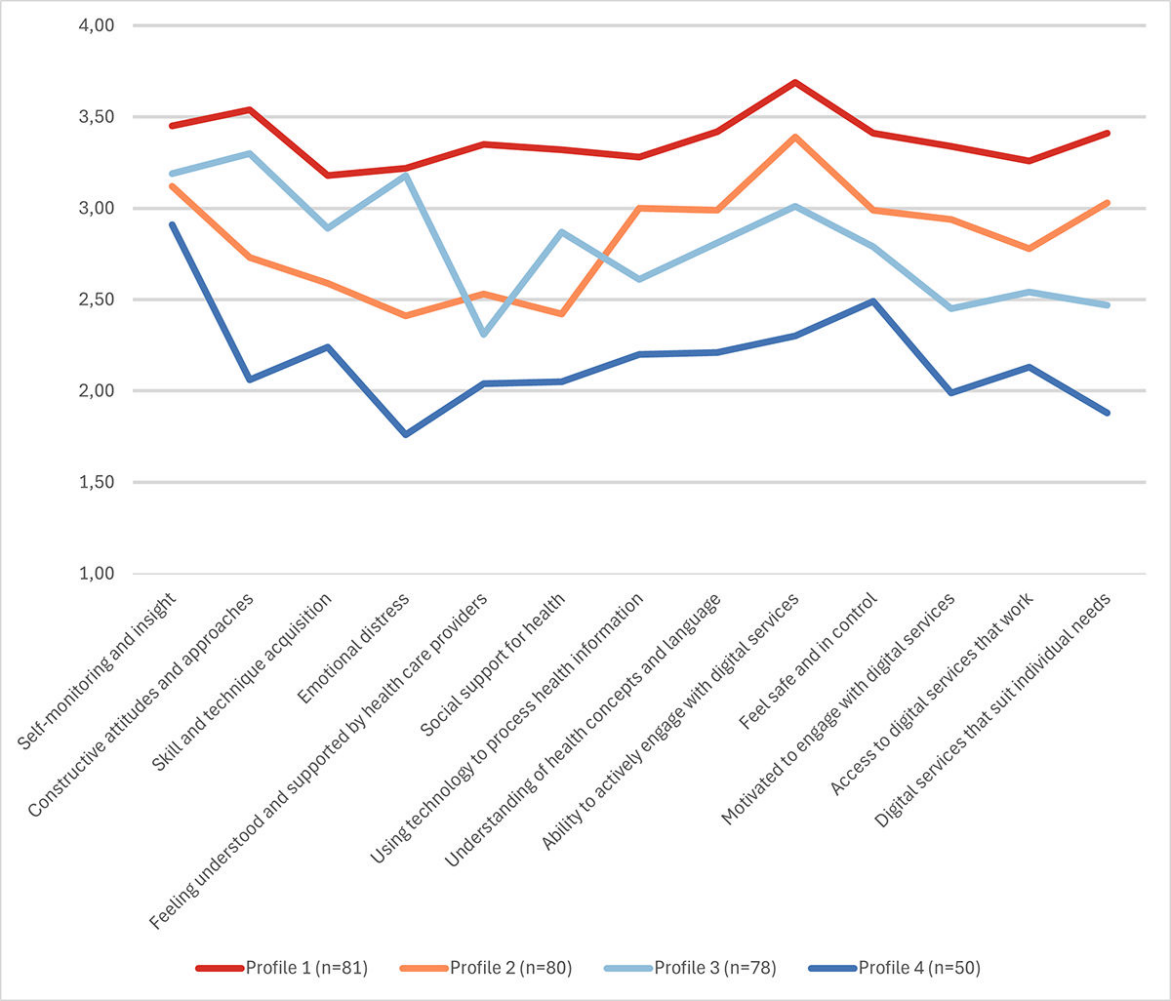
Four profiles of digital health literacy and health technology readiness.

We identified 4 profiles based on respondents’ DHL and health technology readiness (Figure 2).

Profile 1 (81/289, 28%) had a high level of DHL and health technology readiness. They scored high in all READHY dimensions, especially in the *ability to actively engage with digital services* (mean 3.69, SD 0.42) and *constructive attitudes and approaches* (mean 3.54, SD 0.44).

Profile 2 (80/289, 27.7%) had generally high and higher midlevel scores in DHL and health technology readiness. Their scores on DHL ranged between 3.39 (*ability to actively engage with digital services*) and 2.78 (*access to digital services that work*). They had high confidence in their *self-monitoring and insight* (mean 3.12, SD 0.33) but lower midlevel confidence in their abilities to handle *emotional distress* (mean 2.42, SD 0.54).

Profile 3 (78/289, 27%) had generally midlevel DHL and health technology readiness. They had midlevel scores in all DHL dimensions and in the social context. In addition, they had high-level scores in *constructive attitudes and approaches* (mean 3.30, SD 0.45) and in *self-monitoring and insight* (mean 3.19, SD 0.34).

Profile 4 (50/289, 17.3%) had low to lower midlevel DHL and health technology readiness. They did not score high on any READHY dimensions, with the lowest scores in their ability to handle *emotional distress* (mean 1.76, SD 0.51). They had lower midlevel confidence in both dimensions describing their confidence in their social context. However, they had higher midlevel scores on *self-monitoring and insight* (mean 2.91, SD 0.54).

Educational levels varied significantly among the 4 profiles (*P*<.001). Pairwise comparisons indicated that profile 4 had significantly lower educational levels than profiles 1 (*P*<.001) and 2 (*P*=.009).

### DHL and Health Technology Readiness Among Users and Nonusers of DHS

Respondents who used DHS to manage their chronic condition reported higher levels of DHL and health technology readiness than nonusers (Table 3). The significant difference was evident in 6 of the 7 dimensions describing the respondents’ DHL, as well as in both dimensions describing the respondents’ social context. In addition, the difference was significant in *skill and technique acquisition,* one of the 4 dimensions describing individuals’ capabilities to handle their condition and emotional response.

**Table 3.** Means of Readiness and Enablement Index for Health Technology domains among the users (n=188) and nonusers (n=100) of digital health services.

Dimensions	Users, mean (SD)	Nonusers, mean (SD)	*P* value^a^
Self-monitoring and insight	3.23 (0.36)	3.12 (0.50)	.20
Constructive attitudes and approaches	3.06 (0.66)	2.88 (0.79)	.08
Skill and technique acquisition	2.85 (0.50)	2.62 (0.66)	.002
Emotional distress	2.79 (0.72)	2.64 (0.82)	.18
Feeling understood and supported by health care providers	2.71 (0.72)	2.42 (0.90)	.01
Social support for health	2.81 (0.71)	2.54 (0.85)	.02
Using technology to process health information	2.94 (0.50)	2.62 (0.62)	<.001
Understanding of health concepts and language	3.03 (0.50)	2.73 (0.61)	<.001
Ability to actively engage with digital services	3.32 (0.61)	2.93 (0.79)	<.001
Feel safe and in control	3.04 (0.55)	2.84 (0.71)	.06
Motivated to engage with digital services	2.91 (0.58)	2.46 (0.68)	<.001
Access to digital services that work	2.84 (0.55)	2.54 (0.55)	<.001
Digital services that suit individual needs	2.95 (0.30)	2.48 (0.77)	<.001

a *P*<.05 is considered statistically significant.

Users of DHS were significantly younger (*P*=.03) than nonusers. This was the only statistically significant difference identified between the 2 groups in terms of their characteristics. However, we identified significant differences (*P*<.001) in the proportion of DHS users among different profiles. In pairwise comparisons, there were significantly fewer users of DHS among profile 4 compared with profile 1 (*P*<.001) and profile 2 (*P*=.001).

## Discussion

### Principal Findings and Comparison With Prior Research

The key finding of our study was that, although the respondents were fairly to highly confident in their overall DHL and health technology readiness, we identified 4 subgroups with distinct READHY profiles. Each profile had different strengths and weaknesses in their confidence in the 13 READHY dimensions.

Our findings align with those of previous research with the READHY instrument [48,49] and with research using other DHL instruments [17,21]. More specifically, our results indicate that among people with chronic conditions, there are differences in their DHL, suggesting that, in addition to their disease background, other factors are also associated with their DHL, which is consistent with earlier findings [21,55]. However, despite being highly confident in their ability and motivation to manage their condition and emotional response, the respondents reported lower confidence in receiving social support compared with previous research [48,49,56]. Consistent with this, Kuusisto et al [57] reported that people with MS felt that the information obtained from their health care professional was insufficient to facilitate their participation in shared decision-making regarding their care. This potential gap in patient-health care provider communication needs to be addressed to ensure that individuals with chronic conditions receive the support necessary to manage their condition. Further research is needed to determine whether this is a cultural phenomenon or a result of the health care system.

The 4 READHY profiles had different strengths and weaknesses. Profiles 1 and 2, with the highest confidence in their DHL, also had a significantly higher proportion of DHS users compared with profile 4 with the lowest level of DHL, indicating an association between DHL and the use of DHS for managing their chronic condition. However, although profile 1 had high confidence across all dimensions, profile 2 had higher *emotional distress* and lower confidence in their *skill and technique acquisition* to manage their condition and in the social support they received compared with profile 1. This implies that profile 2 could benefit from additional support for managing their health and emotional well-being. Profile 3, with midlevel scores, and profile 4, with low to lower midlevel scores on DHL dimensions and fewer users of DHS, indicated that they could represent individuals who need more support in using DHS. Additionally, profile 4—characterized by the highest emotional distress and the lowest confidence in the social support received compared with the other profiles—may represent individuals who require more support when using DHS to manage their chronic condition and who may therefore be more likely to prefer traditional face-to-face visits.

Our results also indicated that respondents with higher educational levels had significantly higher DHL, supporting the conclusion by Thorsen et al [39] that factors other than general sociodemographic characteristics and having a chronic disease are significant for the level of DHL and health technology readiness. The 4 profiles we identified increase this understanding of the factors related to DHL and health technology readiness. Identifying the various factors that support and hinder each profile’s readiness to use health technology [53] is crucial for developing targeted health technology solutions [57]. Thus, although all 4 profiles reported good confidence in their ability to self-monitor their condition, promoting DHL remains necessary.

Our finding of two-thirds of the respondents having used DHS aligns with research on other chronic conditions [13,37,58]. In addition, DHS could be seen as a channel for frequent contact, as it was used several times a year, even monthly. In comparison, although most participants had face-to-face visits with their health care providers, these visits typically occurred only once or a few times per year. This finding indicates a need to identify the use cases that could be directed to DHS. Furthermore, user-centered development of DHS and the promotion of DHL may support the effective use of DHS.

Users of DHS had higher DHL and health technology readiness than nonusers. In addition, users also had significantly higher confidence in their *social support for health, feeling understood and supported by health care providers*, and *skill and technique acquisition* related to their capabilities to handle their condition. On the basis of these results, more research is required to investigate these relationships. Notably, despite the significant differences between users and nonusers of DHS, the nonusers also had reasonably good DHL and health technology readiness. This could be a result of variation in the level of digitalization among public health care providers, potentially resulting in inequality in access to and opportunities to use DHS.

### Limitations and Recommendations for Future Research

This study has several limitations. First, despite the questionnaires being distributed via patient organizations and a neurology outpatient clinic, the sample size remained limited. Because the total number of individuals who received the questionnaire was unknown, it was not possible to calculate sample estimates. This may affect the generalizability of the findings. Second, because participation was voluntary, our sample may be subject to selection bias, potentially leading to an overrepresentation of DHS users. However, the questionnaires were also distributed in paper format through a neurology outpatient clinic, aiming to reach individuals who might not engage with online platforms. Third, prior computer use was not included as a background variable; thus, the association between DHS use and prior computer use could not be investigated.

In addition, this study was conducted in a single geographical area, reflecting the Finnish health care context, and caution must be exercised when generalizing the findings to other health care systems. Finally, the cross-sectional nature of the study precluded the determination of the evolution of DHL level, health technology readiness, and DHS use, necessitating additional longitudinal research. Further research is also required to elucidate whether the participants’ perceptions of receiving inadequate social support regarding their health are a cultural phenomenon or a result of the health care system. Furthermore, additional research is needed on using the READHY instrument in the Finnish population and other health care systems.

### Conclusions

Our key finding was the identification of 4 subgroups with distinct READHY profiles and different strengths and weaknesses in their DHL and health technology readiness. Understanding these strengths and weaknesses can help in the development of targeted interventions to encourage specific patient groups to take full advantage of DHS. However, this requires the development of processes to facilitate the identification of profiles. Additionally, adequate resourcing of different care channels is needed to meet users’ needs.

We identified 2 profiles with high confidence in their DHL, both of which had a high proportion of DHS users and may possibly represent active users of various DHS. In contrast, the profiles with lower DHL could be more likely to use traditional face-to-face health care services and therefore benefit from the support in using DHS in the management of their chronic conditions. Notably, although all 4 profiles expressed generally high confidence in *self-monitoring and insight*, 3 reported low or medium confidence in *being understood and supported by health care providers*. This underlines the importance of ensuring that all individuals feel understood and supported by their health care providers, regardless of the health care channel they use. Future studies should further explore the phenomenon of not feeling supported by health care providers.

## Supplementary material

10.2196/85625 Multimedia Appendix 1 Mean scores of Readiness and Enablement Index for Health Technology domains and dimensions across participant profiles.

## Abbreviations

DHL: digital health literacy
DHS: digital health services
eHLF: eHealth Literacy Framework
MS: multiple sclerosis
READHY: Readiness and Enablement Index for Health Technology
READHY-FIN: Finnish version of the Readiness and Enablement Index for Health Technology
READHY-SWE: Swedish version of the Readiness and Enablement Index for Health Technology

## References

[1] Eberle C, Stichling S (2021). Clinical improvements by telemedicine interventions managing type 1 and type 2 diabetes: systematic meta-review. J Med Internet Res.

[2] Del Pino R, Díez-Cirarda M, Ustarroz-Aguirre I (2022). Costs and effects of telerehabilitation in neurological and cardiological diseases: a systematic review. Front Med.

[3] Gentili A, Failla G, Melnyk A (2022). The cost-effectiveness of digital health interventions: a systematic review of the literature. Front Public Health.

[4] Alaslawi H, Berrou I, Al Hamid A, Alhuwail D, Aslanpour Z (2022). Diabetes self-management apps: systematic review of adoption determinants and future research agenda. JMIR Diabetes.

[5] Eberle C, Stichling S (2021). Telemetric interventions offer new opportunities for managing type 1 diabetes mellitus: systematic meta-review. JMIR Diabetes.

[6] Kraef C, van der Meirschen M, Free C (2020). Digital telemedicine interventions for patients with multimorbidity: a systematic review and meta-analysis. BMJ Open.

[7] Kuan PX, Chan WK, Fern Ying DK (2022). Efficacy of telemedicine for the management of cardiovascular disease: a systematic review and meta-analysis. Lancet Digit Health.

[8] Lee JJ, Abdul Aziz A, Chan ST (2023). Effects of mobile health interventions on health-related outcomes in older adults with type 2 diabetes: a systematic review and meta-analysis. J Diabetes.

[9] Ma Y, Zhao C, Zhao Y (2022). Telemedicine application in patients with chronic disease: a systematic review and meta-analysis. BMC Med Inform Decis Mak.

[10] Renzi E, Baccolini V, Migliara G (2022). The impact of eHealth interventions on the improvement of self-care in chronic patients: an overview of systematic reviews. Life (Basel).

[11] Choi M (2022). Factors associated with eHealth use among community dwelling older adults. Int J Nurs Pract.

[12] Ramstad KJ, Brørs G, Pettersen TR (2023). eHealth technology use and eHealth literacy after percutaneous coronary intervention. Eur J Cardiovasc Nurs.

[13] Tang J, Howell M, Lee VW (2023). Patients’ perspectives, factors, and patterns of eHealth use in kidney transplant recipients. Kidney Int Rep.

[14] Wong DK, Cheung MK (2019). Online health information seeking and ehealth literacy among patients attending a primary care clinic in Hong Kong: a cross-sectional survey. J Med Internet Res.

[15] Norman CD, Skinner HA (2006). eHealth literacy: essential skills for consumer health in a networked world. J Med Internet Res.

[16] Zangger G, Bricca A, Liaghat B (2023). Benefits and harms of digital health interventions promoting physical activity in people with chronic conditions: systematic review and meta-analysis. J Med Internet Res.

[17] Shiferaw KB, Tilahun BC, Endehabtu BF, Gullslett MK, Mengiste SA (2020). E-health literacy and associated factors among chronic patients in a low-income country: a cross-sectional survey. BMC Med Inform Decis Mak.

[18] Redfern J, Coorey G, Mulley J (2020). A digital health intervention for cardiovascular disease management in primary care (CONNECT) randomized controlled trial. NPJ Digit Med.

[19] Ernsting C, Stühmann LM, Dombrowski SU, Voigt-Antons JN, Kuhlmey A, Gellert P (2019). Associations of health app use and perceived effectiveness in people with cardiovascular diseases and diabetes: population-based survey. JMIR Mhealth Uhealth.

[20] Knitza J, Simon D, Lambrecht A (2020). Mobile health usage, preferences, barriers, and eHealth literacy in rheumatology: patient survey study. JMIR Mhealth Uhealth.

[21] Lee J, Tak SH (2022). Factors associated with eHealth literacy focusing on digital literacy components: a cross-sectional study of middle-aged adults in South Korea. Digit Health.

[22] Schrauben SJ, Appel L, Rivera E (2021). Mobile health (mHealth) technology: assessment of availability, acceptability, and use in CKD. Am J Kidney Dis.

[23] Wu Y, Wen J, Wang X (2022). Associations between e-health literacy and chronic disease self-management in older Chinese patients with chronic non-communicable diseases: a mediation analysis. BMC Public Health.

[24] GBD 2016 Neurology Collaborators (2019). Global, regional, and national burden of neurological disorders, 1990–2016: a systematic analysis for the Global Burden of Disease Study 2016. Lancet Neurol.

[25] (2023). World health statistics 2023: monitoring health for the SDGs, sustainable development goals. World Health Organization.

[26] Heesen C, Solari A (2023). Editorial: Shared decision-making in neurology. Front Neurol.

[27] van der Horst DE, Garvelink MM, Bos WJ, Stiggelbout AM, Pieterse AH (2023). For which decisions is shared decision making considered appropriate? - A systematic review. Patient Educ Couns.

[28] Zhu M, Dong D, Lo HH, Wong SY, Mo PK, Sit RW (2023). Patient preferences in the treatment of chronic musculoskeletal pain: a systematic review of discrete choice experiments. Pain.

[29] Hämäläinen P, Viitala M, Kuusisto H, Ruutiainen J, Soilu-Hänninen M (2024). MyMS: an interface for patient-reported outcomes for Finnish individuals with multiple sclerosis. Int J MS Care.

[30] Ward M, Goldman MD (2022). Epidemiology and pathophysiology of multiple sclerosis. Continuum (Mount Lawley).

[31] Thijs RD, Surges R, O’Brien TJ, Sander JW (2019). Epilepsy in adults. Lancet.

[32] Arsad FS, Syed Soffian SS, Megat Kamaruddin PS (2022). The impact of eHealth applications in healthcare intervention: a systematic review. J Health Res.

[33] Masanneck L, Gieseler P, Gordon WJ, Meuth SG, Stern AD (2023). Evidence from ClinicalTrials.gov on the growth of Digital Health Technologies in neurology trials. NPJ Digit Med.

[34] Kuusisto H, Hämäläinen P, Nurmi H, Kälviäinen R, Soilu-Hänninen M (2025). Envisioning the Future of Health Informatics and Digital Health.

[35] Fitzsimons M, Power K, McCrea Z (2021). Democratizing epilepsy care: utility and usability of an electronic patient portal. Epilepsy Behav.

[36] Voigt I, Benedict M, Susky M (2020). A digital patient portal for patients with multiple sclerosis. Front Neurol.

[37] Vesinurm M, Maunula A, Olli P (2024). Effects of a digital care pathway for multiple sclerosis: observational study. JMIR Hum Factors.

[38] Wenk J, Voigt I, Inojosa H, Schlieter H, Ziemssen T (2024). Building digital patient pathways for the management and treatment of multiple sclerosis. Front Immunol.

[39] Thorsen IK, Rossen S, Glümer C, Midtgaard J, Ried-Larsen M, Kayser L (2020). Health technology readiness profiles among Danish individuals with type 2 diabetes: cross-sectional study. J Med Internet Res.

[40] Jylhä V, Turja T (2023). Health technology readiness: the translation of READHY-FIN questionnaire. FinJeHeW.

[41] Kayser L, Rossen S, Karnoe A (2019). Development of the multidimensional readiness and enablement index for health technology (READHY) tool to measure individuals’ health technology readiness: initial testing in a cancer rehabilitation setting. J Med Internet Res.

[42] Osborne RH, Batterham RW, Elsworth GR, Hawkins M, Buchbinder R (2013). The grounded psychometric development and initial validation of the Health Literacy Questionnaire (HLQ). BMC Public Health.

[43] Osborne RH, Elsworth GR, Whitfield K (2007). The Health Education Impact Questionnaire (heiQ): an outcomes and evaluation measure for patient education and self-management interventions for people with chronic conditions. Patient Educ Couns.

[44] Kayser L, Karnoe A, Furstrand D (2018). A multidimensional tool based on the eHealth literacy framework: development and initial validity testing of the eHealth Literacy Questionnaire (eHLQ). J Med Internet Res.

[45] Hawkins M, Osborne RH (2019). Translation Integrity Procedure (TIP) For the Translation and Cultural Adaptation of Psychometric Questionnaires.

[46] CC BY 4.0: attribution 4.0 international. Creative commons.

[47] Oliveira FK, Oliveira M, Gomes AS, Queiros LM (2018). Identifying user profiles from statistical grouping methods. J Inf Syst Eng Manag.

[48] Rossen S, Kayser L, Vibe-Petersen J, Ried-Larsen M, Christensen JF (2019). Technology in exercise-based cancer rehabilitation: a cross-sectional study of receptiveness and readiness for e-Health utilization in Danish cancer rehabilitation. Acta Oncol.

[49] Nielsen AS, Hanna L, Larsen BF, Appel CW, Osborne RH, Kayser L (2022). Readiness, acceptance and use of digital patient reported outcome in an outpatient clinic. Health Informatics J.

[50] Grove SK, Gray JR, Burns N (2012). The Practice of Nursing Research: Appraisal, Synthesis, and Generation of Evidence.

[51] (2023). The European Code of Conduct for Research Integrity – Revised Edition.

[52] Regulation (EU) 2016/679 of the European Parliament and of the Council of 27 April 2016 on the protection of natural persons with regard to the processing of personal data and on the free movement of such data, and repealing Directive 95/46/EC (General Data Protection Regulation) (Text with EEA relevance). European Union.

[53] Medical Research Act. Ministry of Social Affairs and Health, Finland.

[54] (2019). The ethical principles of research with human participants and ethical review in the human sciences in Finland. https://tenk.fi/sites/default/files/2021-01/Ethical_review_in_human_sciences_2020.pdf.

[55] Madrigal L, Escoffery C (2019). Electronic health behaviors among US adults with chronic disease: cross-sectional survey. J Med Internet Res.

[56] Terp R, Kayser L, Lindhardt T (2021). Older patients’ competence, preferences, and attitudes toward digital technology use: explorative study. JMIR Hum Factors.

[57] Kuusisto H, Apila S, Saranto K (2022). Information provision and quality. A pilot study on shared decision-making in multiple sclerosis. Stud Health Technol Inform.

[58] Tosun AT, Isiklar C, Yildirim M, Coskunsu DK (2024). e-Health literacy status of individuals with multiple sclerosis in Turkey. Telemed J E Health.

