# Supplementary material for: Digital Health Literacy and Health Technology Readiness Among People With Epilepsy or Multiple Sclerosis: Cross-Sectional Study
Source: JMIR Hum Factors. 2026 Mar 5;13:e85625. doi: 10.2196/85625 (PMC12978907; doi:10.2196/85625)
Supplement: Multimedia Appendix 1 [file humanfactors-v13-e85625-s001.docx]

Multimedia Appendix 1

Table S1. Mean scores of READHY domains and dimensions across participant profiles

| **READHY domains and dimensions** | **All  (N = 289)**  **mean (SD)** | **Profile 1 (n = 81) mean (SD)** | **Profile 2**  **(n = 80) mean (SD)** | **Profile 3 (n = 78) mean (SD)** | **Profile 4 (n = 50) mean (SD)** |
| --- | --- | --- | --- | --- | --- |
| Individuals' capabilities to manage their condition and emotional response | 2.80 (.252) | 3.35 (.303) | 2.71 (.365) | 3.14 (.484) | 2.24 (.332) |
| Self-monitoring and insight | 3.19 (.416) | 3.45 (.339) | 3.12 (.317) | 3.19 (.335) | 2.91 (.538) |
| Constructive attitudes and approaches | 3.00 (.706) | 3.54 (.446) | 2.73 (.463) | 3.30 (.448) | 2.06 (.571) |
| Skill and technique acquisition | 2.78 (.565) | 3.18 (.501) | 2.59 (.443) | 2.89 (.368) | 2.24 (.580) |
| Emotional distress | 2.74 (.755) | 3.22 (.536) | 2.41 (.539) | 3.18 (.432) | 1.76 (.507) |
| Individuals' social context | 2.67 (.683) | 3.33 (.436) | 2.47 (.356) | 2.59 (.519) | 2.04 (.616) |
| Feeling understood and supported by healthcare providers | 2.61 (.820) | 3.35 (.586) | 2.53 (.698) | 2.31 (.638) | 2.04 (.760) |
| Social support for health | 2.72 (.765) | 3.32 (.601) | 2.42 (.576) | 2.87 (.612) | 2.05 (.704) |
| Users' attributes | 2.98 (.540) | 3.46 (.303) | 3.12 (.365) | 2.81 (.296) | 2.24 (.420) |
| Using technology to process health information | 2.83 (.563) | 3.28 (.418) | 3.00 (.373) | 2.61 (.394) | 2.20 (.496) |
| Understanding of health concepts and language | 2.92 (.558) | 3.42 (.364) | 2.99 (.428) | 2.81 (.365) | 2.21 (.379) |
| Ability to actively engage with digital services | 3.19 (.700) | 3.69 (.399) | 3.39 (.518) | 3.01 (.530) | 2.30 (.667) |
| Intersection between users and technologies | 2.86 (.528) | 3.38 (.355) | 2.96(.311) | 2.62 (.337) | 2.24 (.383) |
| Feel safe and in control | 2.97 (.615) | 3.41 (.511) | 2.99 (.391) | 2.80 (.579) | 2.49 (.652) |
| Motivated and engaged with digital services | 2.75 (.649) | 3.34 (.424) | 2.94 (.435) | 2.45 (.453) | 1.99 (.432) |
| Users’ experiences of systems | 2.76 (.592) | 3.34 (.449) | 2.91 (.351) | 2.51 (.369) | 2.00 (.616) |
| Access to digital services that work | 2.73 (.566) | 3.27 (.466) | 2.78 (.387) | 2.54 (.395) | 2.13 (.417) |
| Digital services that suit individual needs | 2.79 (.715) | 3.41 (.497) | 3.03 (.428) | 2.47 (.449) | 1.88 (.493) |
